# The patient education strategy “learning and coping” improves adherence to cardiac rehabilitation in primary healthcare settings: a pragmatic cluster-controlled trial

**DOI:** 10.1186/s12872-022-02774-8

**Published:** 2022-08-08

**Authors:** Charlotte Gjørup Pedersen, Claus Vinther Nielsen, Vibeke Lynggaard, Ann Dorthe Zwisler, Thomas Maribo

**Affiliations:** 1 Department of Public Health, Aarhus University, Aarhus, Denmark; 2 DEFACTUM, Central Denmark Region, Aarhus, Denmark; 3 REHPA, The Danish Knowledge Centre for Rehabilitation and Palliative Care, Odense University Hospital, Nyborg, Denmark; 4 Department of Clinical Research, University of Southern Denmark, Odense, Denmark; 5 Cardiovascular Research Unit, Department of Cardiology, Gødstrup Hospital, Herning, Denmark; 6 Department of Clinical Social Medicine and Rehabilitation, Gødstrup Hospital, Herning, Denmark; 7 Department of Cardiology, Odense University Hospital, Odense, Denmark

**Keywords:** Cardiac rehabilitation, Primary healthcare settings, Patient education strategy, Adherence, Completion, Self-management

## Abstract

**Background:**

Adherence and completion of programmes in educational and physical exercise sessions is essential in cardiac rehabilitation (CR) to obtain the known benefits on morbidity, mortality, risk factors, lifestyle, and quality of life. The patient education strategy “Learning and Coping” (LC) has been reported to positively impact adherence and completion in a hospital setting. It is unknown if LC has impact on adherence in primary healthcare settings, and whether LC improves self-management. The aim of this pragmatic primary healthcare-based study was to examine whether patients attending CR based on LC had a better adherence to patient education and physical exercise, higher program completion rate, and better self-management compared to patients attending CR based on a consultation program Empowerment, Motivation and Medical Adherence (EMMA).

**Method:**

A pragmatic cluster-controlled trial of two types of patient education LC and EMMA including ten primary healthcare settings and 514 patients (LC, n = 266; EMMA, n = 248) diagnosed with ischaemic heart disease discharged from hospital and referred to CR between August 1, 2018 and July 31, 2019. Adherence was defined as participation in ≥ 75% of provided sessions. Completion was defined as patients attended the final interview at the end of the 12-weeks programme. Patient Activation Measure (PAM) was used to obtain information on a person's knowledge, skills and confidence for self-management. PAM questionnaire was completed at baseline and 12-weeks follow-up. Multiple and Linear regression analyses adjusted for potential confounder variables and cluster effect were performed.

**Result:**

Patients who followed CR based on LC had a higher adherence rate to educational and physical exercise sessions compared to patients who followed CR based on EMMA (p < 0.01). High-level of completion was found at the end of CR with no statistically significant between clusters (78.9% vs. 78.2%, p > 0.05). At 12-weeks, there was no statistical differences in PAM-score between clusters (p > 0.05).

**Conclusion:**

This study indicates that the LC positively impacts adherence in CR compared to EMMA. We found non-significant difference in completing CR and in patient self-management between the two types of patient education. Future studies are needed to investigate if the higher adherence rate achieved by LC in primary healthcare settings translates into better health outcomes.

**Supplementary Information:**

The online version contains supplementary material available at 10.1186/s12872-022-02774-8.

## Background

Cardiac rehabilitation (CR) is a Class I recommendation in CR guidelines for patients with ischaemic heart disease (IHD) [1–3] as it increases quality of life and reduces mortality and cardiovacular morbidity [4–7]. Health behaviour change and patient education to develop self-management skills remains fundamental to all recommened components of CR [2]. Adherence in CR is poor worldwide and varies from 36.7% to 84.6% [8, 9]. Several risk factors entail non-attendance and dropout from CR such as low socioeconomic status and comorbidity [8]. Patient education empower and prepare cardiac patients to increase knowledge and promote health behavior change [10, 11]. Therefore, it is necessary to examine the impact of patient education on adherence and which types produces the best benefit for patients with IHD.

Patient education is any set of planned educational activities designed to improve patients’ knowledge and health behaviours [12]. In CR guidelines, several educational topics are listed as important to enable patients to learn about their condition and improve self-management [1–3]. However, the guidelines only advice on what to teach, not how it should be done and no special type of patient education is promoted. In Central Denmark Region, the local guideline for cross-sectoral collaboration in CR refer to two different evidence-based models of patient education including Learning and Coping strategy (LC) and consultation program Empowerment, Motivation and Medical Adherence (EMMA) [13].

LC builds on inductive teaching with high involvement of the participants and participation of experienced former cardiac patients. In LC, experienced patients plan sessions as well as act as co-educators telling narratives in the education sessions and evaluate the sessions in collaboration with healthcare providers [14]. EMMA builds on a consultation program with dialog tools developed to patients with type 2 diabetes [15]. These dialogue tools are used to explore specific challenges for medication adherence, perform medical review and facilitate interactive learning and goal-setting and action-planning processes [15].

LC and EMMA have been used for several years in primary healthcare settings without knowing if one is superior to the other. In a hospital setting, LC has shown increased adherence and completion to CR [16]. So far, it is unclear whether LC in primary healthcare settings perform similar results. This is relevant since primary healthcare settings provide non-pharmacological phase II cardiac rehablitation in Denmark; this phase encompasses the immediate post discharged period [17]. If LC has an increased adherence and completion rate compared to EMMA, LC are to be recommended. This is especially important if more patients with low socioeconomic status would complete as they also often are at higher risk for a new cardiac event.

The aim of this study was to examine whether patients who followed CR based on the patient education strategy LC had a better adherence to patient education and physical exercise, higher program completion rate, and better self-management compared to patients who followed CR based on EMMA.

## Method

This is a pragmatic primary healthcare-based cluster-controlled trial of two types of patient education used in CR. The trial was reported and conducted in accordance with the Consolidated Standards of Reporting Trials (CONSORT) extension for randomized trials of non-pharmacologic treatment [18].

### Participants

Patients [18 years or older] diagnosed with IHD, discharged from a hospital and referred to CR in ten primary healthcare settings in Central Denmark Region between August 1, 2018 and July 31, 2019 were included. All primary healthcare settings in Central Region Denmark [18 centres] were invited to participate; ten participated, all delivering CR in accordance with the national guidelines [17]. LC was used in six settings, and EMMA was used in four settings. Patients were recruited at the initial interview with a healthcare provider.

### The content of CR in the two clusters

The content of CR in primary healthcare settings has not previously been described systematically. To describe CR, we used the Template for Intervention Descriptions and Replication (TIDieR) [19]. The 12 items in TIDieR were described by interviewing all participating CR teams. Patients were referred to CR by a cardiologist after examination and risk assessment. CR in the the two clusters is a 12-weeks programme comprising the core components of CR assessment, risk factor management, structured exercise training, patient education and psychosocial counselling [2]. Each setting had an assigned CR team of nurses and physiotherapists delivering CR. Asssessment were done at the hospital at discharge and again at the beginning of CR at an one hour consultation [14].

All primary healthcare settings delivered 24 sessions of structured exercise training sessions with focus on aerobic exercise training. In 8 of 10 settings the exercise training session lasted 60 min in the two remaining session lased 70–75 min, psychosocial counselling was delivered if needed, the intervention is comparable to other European contries [20]. Patient education differed between the clustes as decribed below. The 12-weeks CR endend with a consultation on future coping strategies to use after CR. Further, patients were controlled at the hospital and a report was send to the GP [21].

Patient education in all primary healthcare settings focus on empowering the patient to be able to perform self-care by; (1) exploring the patients’ reasons for the visit and concerns, (2) seeking a holistic understanding of the patient, (3) finding common ground on the problem in question and agreement on management, (4) enhancing prevention and health promotion, and (5) enhancing a continuing relationship between the patient and the healthcare provider [22]. In addition, all healthcare settings applied Motivational Interviewing [23], a theory used by providers to explore the patient's ambivalence, enhance motivation and commitment for change and support the patient's autonomy to change.

#### LC

LC builds on inductive teaching with high involvement of the participants [14]. It aims for people with chronic disease to improve their coping, social skills and health behaviour. Experienced former CR patients participated as co-educators and narrators in all of the education sessions. The experienced patients narratives were incorporated as real-life cases. Each week, an one-hour assigned evaluation meeting was held by the nurse, physiotherapist and experienced patient. The team discussed and reflected on the sessions and needs of the individual patients and they evaluated successes and aspects of improvement. Additionally, the team made plans for the following week’s education and training sessions—including a situational, reflective and inductive approach to include the individual patient’s needs and concerns. Healthcare providers and experienced patients had been educated in the LC principles; a two-day course with four moduls for healthcare providers and two moduls for experienced patients. The four moduls included (1) the initial patient interview, (2) the coping process, (3) collaboration between experienced patients and healthcare providers and (4) planning education [14]. Patient educating in the 6 LC settings were delivered at a mean of 8.6 sessions mean time for each session 105 min, see Table 1.

#### EMMA

EMMA builds on the five-step empowerment model of goal setting, combined with supportive individual learning processes to promote action competencies based on individual needs and resources [15]. Patient engagement is facilitated using dialogue tools to support (1) exploration of individuals patients’ challenges and needs, (2) patient education and (3) collaborative goal-setting processes [15]. The team also used a toolkit for group-based education called the NExtEDucation [24]. The toolkit consisted of 24 exercises developed to support health educators in ensuring that patients’ experiences and concerns are the centre of attention [24]. The exercises are based in two theoretical health education models; “The Balancing Person” [25] and “The Health Education Juggler” [26]. Healthcare providers has been educated in the EMMA principles. Patient educating in the 4 EMMA settings were delievered at a mean of 7.25 sessions mean time for each session 90 min, see Table 1. In this cluster two healthcare settings started recruiting patients three and six months later than the other healthcare settings due to change of leadership and logistical challenges in the recruitment process.

**Table 1:** Educational and physical exercise sessions including time consumption per session provided in the CR programme

	LC						EMMA^1^			
	Healthcare settings						Healthcare settings			
	1	2	3	4	5	6	7	8	9	10
Number of education sessions	9	8	9	8	12	6	5	8	8	8
Time consumption per education session (in minutes)	90	90	75	90	75	120	120	90	90	120
Number of physical exercise sessions	24	24	24	24	24	24	24	24	24	24
Time consumption per physical exercise session (in minutes)	70	60	60	75	60	60	60	60	60	60

^1^ Two healthcare settings started recruiting patients three and six months later than the other healthcare settings due to change of leadership and logistical challenges in the recruitment process

### Data sources

The “Cardiac Rehabilitation in Primary Healthcare Settings” is a mandatory online quality improvement database on CR established by regional health authorities in Central Denmark Region and used to obtain data for primary and secondary outcomes and covariates in this study (described below) [27]. At baseline and at the end of the 12-weeks programme, patients completed the 13-items Patient Activation Measure (PAM) to measure a person's knowledge, skills and confidence for self-management [28–30]. At baseline, Hospital Anxiety and Depression Scale (HADS) was used to asses states of anxiety and depression and consist of 14 items—seven concerning anxiety and seven concerning depression. A subscale score > 8 denotes anxiety or depression [31, 32]. Patients answered the questionnaires electronically or by telephone-two reminders were sent.

### Primary outcome

Participation in patient education and physical exercise were recorded in the database; this was used as numerator to calculate each patient’s adherence to patient education and physical exercise; the denominator was provided sessions at enrolled healthcare setting (Table 1). Adherence for each patient was calculated in percentage and grouped ≥ 75% or < 75% of the provided sessions, a cut-off of ≥ 75% has been defined as high-level adherence in CR programmes [16, 33].

### Secondary outcomes

Completion of CR was defined as whether patients attended the final interview at the end of the 12-weeks programme as recorded in the database. PAM responses were used to state patients’ level of self-management; (1) believing the patient role is important, (2) having the confidence and knowledge necessary to take action, (3) actually taking action to maintain and improve one's health, and (4) staying the course even under stress [28].

### Covariates

Lower adherence has independently been predicted by low socioeconomic status, current smoking, non-surgical diagnosis, exercise-limiting comorbidities and lower age [34]. Adults with moderate depression, anxiety or stress are significantly less likely to adhere to CR compared with persons with normal-mild symptoms [35]. In this study we included; sex, age, socioeconomic status (living alone, level of education and employment status), comorbidities (using Charlson Comorbidity Index), smoking status and level of depression and anxiety (using HADS). Subgroups of each covariates are shown in Table 2.

**Table 2:** Baseline characteristics and HADS—anxiety and depression score for patients in both clusters

	LC (n = 266)		EMMA (n = 248)	
Male gender, n (%)	210	(78.9%)	188	(75.8%)
Age, mean (SD)	64.5	(10.0)	63.5	(10.3)
Living alone, n (%)	57	(21.4%)	65	(26.2%)
Level of education*, n (%)				
None, short courses and other^1^	75	(28.2%)	47	(18.9%)
Skilled worker	106	(39.8%)	95	(38.3%)
Higher education (until 4 years)	69	(26.0%)	89	(35.9%)
Higher education (more than 4 years)	16	(6.0%)	17	(6.9%)
Employed, n (%)	124	(46.6%)	116	(46.8%)
*Charlson comorbidity index*				
0	180	(67.6%)	171	(68.9%)
1	42	(15.8%)	34	(13.7%)
>2	44	(16.6%)	43	(17.4%)
*Smoking status, baseline, n (%)*				
Yes	40	(15.0%)	36	(14.5%)
Ex-smoker (> 6 months)	128	(48.1%)	106	(42.7%)
Ex-smoker (< 6 months)	22	(8.3%)	23	(9.3%)
Never^2^	76	(28.6%)	83	(33.4%)
Mean, HADS-anxiety score (SD)^3^	3.5	(3.4)	3.6	(3.4)
Mean, HADS—depression score (SD)^4^	2.9	(3.3)	3.0	(3.2)

* p = 0.03. ^1^ Including unknown LC, n = 17. EMMA, n = 14

^2^ Including unknown LC, n = 9. EMMA n = 13

^3,4^ N = 453. Missing LC n = 42. EMMA n = 19

### Panel of healthcare providers

A panel of healthcare providers was formed to validate the relevancy and transferability of our findings to practice. Healthcare providers from all participating centres took part in the discussion [36]. The panel met three times before the study to discuss primary outcome and the use of questionnaires. After the study period the panel met again where the results were presented. The result from the last meeting were used to structure the discussion.

### Sample size

The study consecutively included all referred patients to CR in ten primary healthcare settings in Central Denmark Region between August 1, 2018 and July 31, 2019, one centre included from October 1 2018 and one centre from January 1, 2019 due to logistical challenges at the centres. Before the study was initiated, we performed a sample size caluculation, which supported that one year inclusion of patients including at least 312 patients per cluster would achieve a power of at least 80% with respect to find a statistically significant difference in health-related quality of life (HRQol) between the two clusters. Sample size of the trial was based on our secondary outcome HRQol, the study was not dimensioned to detect a specific difference in completion proportions. Rather, from this perspective completion proportions are the results of an observational study, and consequently we will report estimates and confidence intervals indicating the precision of our estimates, but no p-values. This includes our estimate of risk difference and odds ratio.

### Statistics

Lost to follow-up was presented with gender and age and differences were estimated with relative risk (gender) and t-test (mean age). Adherence for each patient was calculated in percentage, patients who did not want to participate in patient education (12 patients in LC and 44 patients in EMMA) and physical exercise (7 patients in LC and 15 patients in EMMA) were grouped as < 75%. PAM responses were displayed between 1 and 100 [29]. Multiple logistic regression was used to examine the primary outcome and completion rate in the secondary outcome. Linear regression was used to estimate differences in PAM-score between clusters and from baseline to follow-up [12-week] within clusters. Results from the two regressions models were presented as odds ratios (OR) and coefficients with 95% confidence intervals (CI). Covariates were examined as possible confounders. Sensitivity analysis were performed to determine whether missing HADS responses (13.9% (n = 37) in LC and 9.6% (n = 24) in EMMA) would affect the adjusted regression results. The literature was used to state the worse-case scenario [37, 38]; 20% scored low symptoms (score 0–7), 60% scored moderate symptoms (score 8–10) and 20% scored high symptoms (score 11–21) and tested in the regression models. The results are shown in Additional file 1 and 2. All analyses were performed using STATA version 16 (StataCorp LP, College Station, TX, USA).

## Results

**Fig. 1:**
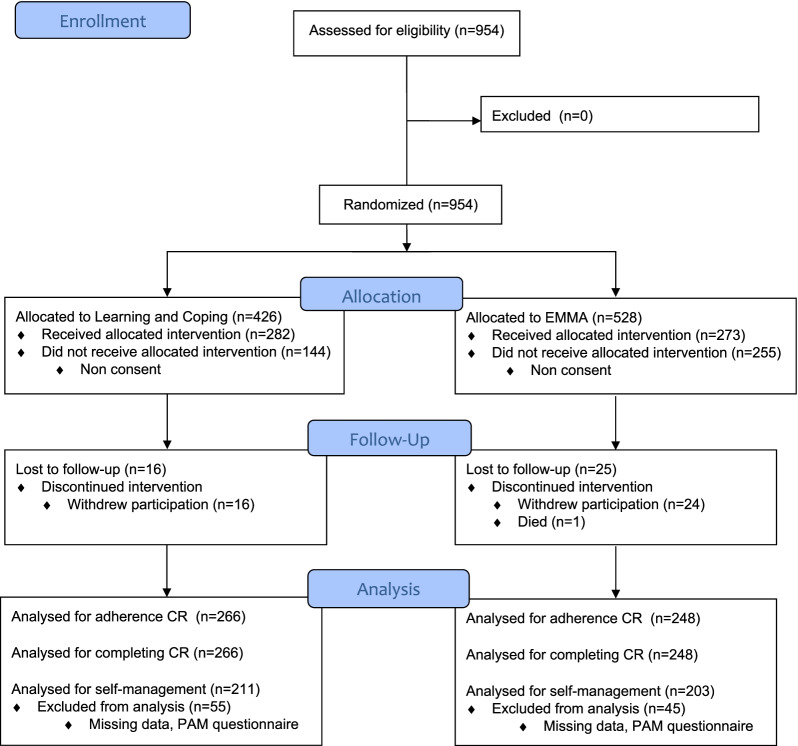
Flow diagram of study population. Note. Two healthcare settings with EMMA started recruiting patients three and six months later than the other healthcare settings due to change of leadership and logistical challenges in the recruitment process. Therefore, n = 244 of patients were not recruited (not shown)

In total, 954 patients were recruited, of these 58.1% (n = 555) patients consented to participate in this study; 66.1% (n = 282) in LC and 51.7% (n = 273) in EMMA. (Fig. 1).

### Lost to follow-up

In total, 7.3% (n = 41) out of 555 persons was lost to follow-up. Fourty persons chose to withdraw consent and one person died. Only gender and age were availble for this group. We found no statistically significant difference between gender (baseline: male n = 423, female n = 232. Lost to follow-up: male n = 25, female n = 16), p > 0.05. A statistically significant difference was found in age (baseline; mean 64.61 years (standard diviation (SD) = 10.35. Lost to follow-up, mean 71.48 years (SD = 9.90), p = 0.000.

### Study population

Baseline characteristics and HADS—anxiety and depression score for patients in both clusters, these figures are presented in Table 2. Only differences between the two clusters were found in level of education (p = 0.03).

### Primary outcome

The proportion of patients who participated in ≥ 75% of educational and physical exercise sessions was 63.1% (n = 168, N = 266) in LC and 38.3% (n = 95, N = 248) in EMMA (figures shown in note in Table 3). Thus, patients who followed CR based on LC had a significant higher adherence rate to provided programme including both educational and physical exercise sessions compared to patients who followed CR based on EMMA (p < 0.05). We found identical results in the adjusted analysis reduced to the number of patients who had answered the HADS questionnaire (Table 3, adjusted OR = 3.62) and performed sensitivity analysis (Additional file 1, adjusted OR = 2.94). The sensitivity analysis only differed from Table 3 in patient education, showing no difference in adherence to ≥ 75% of patient education between the two clusters (Additional file 1, adjusted OR = 2.74).

**Table 3:** Difference between LC and EMMA, patients who adhered ≥ 75% to patient education and physical exercise

		Crude (N = 453)		Adjusted^4^ (N = 453)	
		OR	95% CI	OR	95% CI
Patient education and physical exercise ≥ 75%^1^	LC	3.42	1.63;7.18	3.62	1.56;8.40
	EMMA	1 (ref)	–	1 (ref)	–
Patient education ≥ 75%^2^	LC	3.04	0.99;9.31	3.33	1.01;10.95
	EMMA	1 (ref)	–	1 (ref)	–
Physical exercise ≥ 75%^3^	LC	2.68	1.42;5.06	2.89	1.44;5.77
	EMMA	1 (ref)	–	1 (ref)	–

^1^ ≥ 75% of provided patient education and physical exercise LC 63.1% (n = 168) EMMA 38.3% (n = 95)

^2^ ≥ 75% of provided patient education sessions LC 59.7% (n = 159) EMMA 37.5% (n = 93)

^3^ ≥ 75% of provided physical exercise sessions LC 68.7% (n = 183) EMMA 51.6% (n = 128).

^4^ Adjusted for sex, age, socioeconomic status (living alone, level of education and employed), comorbidities—using Charlson Comorbidity Index, smoking status and level of depression and anxiety (HADS—baseline)

The number of provided patient education sessions varies between the two clusters. Likewise, time spent on each patient education varies; in average, LC used 89.71 min (95% CI 87.70;91.73 min), while EMMA used in average 104.51 min (95% CI 102.63;106.39 min) (difference _(LC-EMMA)_ = − 14.79 min, p < 0.001). All healthcare settings provided 24 physical exercise session, but time spent on these sessions varied between clusters; in average, LC used 63.47 min (95% CI 62.77;64.17 min), while EMMA used 60.00 min (95% CI 60.00;60.00 min) (difference _(LC-EMMA)_ = 3.47 min, p < 0.001).

### Secondary outcome

#### Completing CR

In total, 78.9% (n = 210) in LC and 78.2% (n = 194) in EMMA completed CR. There were no difference in completion rate between the clusters; we found identical results in the adjusted analysis reduced to the number of patients who had answered the HADS questionnaire (Table 4, adjusted OR = 1.32) and performed sensitivity analysis (Additional file 2, adjusted OR = 1.13).

**Table 4:** Odds ratio for completing CR between the two clusters

		Crude (N = 453)		Adjusted^1^ (N = 453)	
		OR	95% CI	OR	95% CI
Completing	LC	1.22	0.63;2.32	1.32	0.70;2.49
	EMMA	1 (Ref)	–	1 (Ref)	–

^1^ adjusted for sex, age, socioeconomic status (living alone, level of education and employed), comorbidities—using Charlson Comorbidity Index, smoking status and level of depression and anxiety (HADS—baseline)

#### Self-management

The PAM 12-weeks response rates were 79.3% (n = 211) in LC and 83.0% (n = 206) in EMMA. Table 5 shows that mean PAM-score was 64.47 in LC and 64.95 in EMMA which is equivalent to PAM-level 3. The adjusted coefficients showed no statistically significant difference in PAM-score 12-week between the two clusters (Table 5). In both clusters PAM-score improved from baseline to 12-week (Table 5). Moreover, 26.6% (n = 71) of patients in LC and 29.8% (n = 74) in EMMA were categorised with lower PAM-score at 12-week than baseline. Despite a decrease in PAM-score, the majority of patients were categorised in the same PAM-level (LC: 42.2% (n = 30), EMMA: 39.1% (n = 29). The remaining patients ended one PAM-level lower than they reported at the start of CR, this development was equal in both clusters. Furthermore, 27.0% (n = 57) in LC and 29.1% (n = 60) in EMMA were grouped in PAM-level 1 and 2 at the end of CR programme.

**Table 5:** Mean PAM-score 12-week and regression results (coefficients) between and within the two clusters

	N = 414^1^	Mean PAM-score (SD)		Crude coefficient	95% CI	Adjusted coefficient^2^	95% CI
12 week between clusters	LC (n = 211)	64.47	(14.03)	− 0.48	− 3.62;2.66	− 0.13	− 2.60;2.33
	EMMA (n = 203)	64.95	(14.82)	Ref	–	Ref	–
Within LC between baseline and 12 week	LC 12 week	64.47	(14.03)	0.54	0.44;0.64	0.52	0.46;0.58
	LC baseline	61.20	(13.06)	Ref	–	Ref	–
Within EMMA between baseline and 12 week	EMMA 12 week	64.95	(14.82)	0.62	0.43;0.80	0.64	0.45;0.84
	EMMA baseline	62.65	(12.70)	Ref	–	Ref	–

^1^ Three patients excluded due to missing HADS baseline—to include HADS in the regression model

^2^ sex, age, socioeconomic status (living alone, level of education and employed), comorbidities—using Charlson Comorbidity Index, smoking status and level of depression and anxiety (HADS—baseline)

#### Panel of healthcare providers

Preliminary results from the study were presented for the panel, this did not result in further analyses or changes, but the panel had a thorough discussion on implementation and important aspects are presented in the discussion.

## Discussion

Results showed that interventions using LC had an adjusted three-fold higher effect on adherence to educational and physical exercise sessions in CR than EMMA and patients in both patient education strategies had an equally high completion rate. We found no significant differences in patient self-management between the two patient education strategies.

### Adherence and completing CR

Adherence to CR is important to empower and prepare patients with cardiac disease to manage their health and health care [10, 11, 39]. Also, to obtain the known benefits on morbidity, mortality, risk and lifestyle profile and quality of life [4–7]. We found that LC improved adherence to CR in primary healthcare settings; this result is in line with a previous result from a hospital setting [16]. Active involvement of experienced patients is a key element of LC and may explain the improved adherence. This is also in line with a review study which has shown that experienced patients promote and maintain adherence to programs [40].

Our results have been discussed with a panel of healthcare providers from the ten healthcare settings. They experienced that patient education and physical exercise scheduled for the same day improve patients adherence. Likewise, the patients' age and functional capacity is important to consider in planning the programme to maintain patients' adherence and completion. These clinical experiences are relevant as patient-reported reasons for non-adherence have been stated as; did not need the cardiac rehablitation trajectory, the program was not personal enough, content of CR program was not as expected, and intensity of the CR program was too burdensome [41]. Low adherence may also be due to comorbidities and patient who “already exercise regularly” [41]. Patient-reported information on non-adherence was not available, but it would be relevant to clarify why half or less of the patients in LC and EMMA adhered ≥ 75% of a full programme. Like, to discuss why our results in adherence to ≥ 75% in patient education or physical exercise (37.6% to 59.7%) are significantly lower than results from a similar study at hospital-level, where about 70% to 80% participated in ≥ 75% of the sessions [16]. However, it is important to note that the hospital study included patients with IHD and heart failure, and subgroup analyzes showed that patients with IHD were less likely to adhere in 75% patient education and physical exercise than patients with heart failure [16]. Although patients did not adhere to the full programme, our results showed a high completion rate in both clustres. This result is in line with hospital-level completion rates where LC was compared with usual CR [16].

### Self-management

We found no statistically significant difference in PAM-scores between the clusters. Other interesting findings are that a significant proportion of patients were categorised with PAM-level 1 and 2 at the end of the 12-weeks CR, like several patients had a lower PAM-level at the of the 12-weeks CR. Low PAM-levels need attention, as chronically ill patients with low stages of PAM are at an increased risk for hospitalisation and emergency room utilisation [42]. Our results show that there is a need for further investigation in patients with low self-management after a 12-weeks program and whether this group need a more focused intervention. Using PAM as a screening tool may induce a more differentiated rehabilitation program and improve equality, as patients with higher PAM-level are significantly more likely to exercise regularly, follow a low-fat diet, eat more fruits and vegetables, and not smoke [28].

### Strengths and limitations

The strength in this study was the use of a well-implemented database (Cardiac Rehabilitation in Primary Healthcare Settings) from which we obtained data from ten out of 18 primary healthcare settings. Furthermore, it is a strength that TIDieR has been used and thus entailed a systematic content description of CR described the differences and similarities in the two types of patient education; TIDieR provides the opportunity for other professionals and researchers to reproduce LC in new studies.

It seems a strength that healthcare providers recruited patients at the first interview, as healthcare providers are considered a trusted group, thus more patients’ have given consent to participate. On the other hand, the recruitment process could also limit patients’ participation, as the healthcare providers in this study was inexperienced in recruiting patients for research studies. Patients reasons for not participating in the study were not investigated, but discussed with a panel of healthcare providers, who highlighted following reasons: severity of illness, do not want to answer questionnaires, participate in other research projects or do not have time to participate. These challenges including delayed recruitment in two healthcare settings have led to a lower sample size than expected.

It may be questioned whether this study population is representative for patients referred to CR in primary healthcare settings, as patients at baseline reported low symptoms of anxiety and depression and high PAM-level [3 and 4] which may have created a healthy user bias. Furthermore, recall bias may occur, as several patients who followed LC and EMMA were reminded to answer the questionnaires.

The sample size calculation was not based on our primary outcome may be a limitation. However, it may be a strength as we used the secondary outcome, which is the most relevant for the patients namely HRQol. Furthermore, the small variation in the number of provided patient education sessions entailed adjustment for cluster effect in the regressions analysis.

## Conclusion

This pragmatic study finds that LC positively impacts adherence in cardiac rehablitation compared to EMMA, as adherence is important for outcome of CR, LC is to be recommended. We found no significant differences in completing CR and patient self-management between the two types of patient education. Future follow-up studies are needed to investigate if the higher attendance rate achieved by LC in primary healthcare settings translates into better health outcomes.

## Supplementary Information

**Additional file 1**. Sensitivity analysis including missing HADS responses (LC n=42, EMMA n=19): 20% scored low symptoms (score 0-7), 60% scored moderate symptoms (score 8-10) and 20% scored high symptoms (score 11-21).

**Additional file 2**. Sensitivity analysis including missing HADS responses (LC n=42, EMMA n=19): 20% scored low symptoms (score 0-7), 60% scored moderate symptoms (score 8-10) and 20% scored high symptoms (score 11-21).

## Data Availability

All data and material collected during this study are available from the corresponding. author upon reasonable request.

## Abbreviations

CR: Cardiac rehabilitation
LC: Learning and coping strategy
EMMA: Empowerment, motivation and medical adherence
PAM: Patient activation measure
IHD: Ischaemic heart disease
OR: Odds ratio
CI: Confidence interval
SD: Standard deviation
