# Supplementary material for: The patient education strategy “learning and coping” improves adherence to cardiac rehabilitation in primary healthcare settings: a pragmatic cluster-controlled trial
Source: BMC Cardiovasc Disord. 2022 Aug 8;22:364. doi: 10.1186/s12872-022-02774-8 (PMC9361528; doi:10.1186/s12872-022-02774-8)
Supplement: Supplementary file 1 — Additional file 1. Sensitivity analysis including missing HADS responses (LC n=42, EMMA n=19): 20% scored low symptoms (score 0-7), 60% scored moderate symptoms (score 8-10) and 20% scored high symptoms (score 11-21). [file 12872_2022_2774_MOESM1_ESM.docx]

Additional file 1

Table with results from the sensitivity analysis including worst-case scenario, which was tested in a multiple regression model.

|  |  | Crude (N=514) | | Adjusted^4^ (N = 514) | |
| --- | --- | --- | --- | --- | --- |
|  |  | OR | 95% CI | OR | 95% CI |
| Patient education and physical exercise > 75%^1^ | LC | 2.76 | 1.48;5.13 | 2.94 | 1.41;6.14 |
|  | EMMA | 1 (ref) | - | 1 (ref) | - |
| Patient education > 75%^2^ | LC | 2.47 | 0.87;7.03 | 2.74 | 0.91;8.26 |
|  | EMMA | 1 (ref) | - | 1 (ref) | - |
| Physical exercise > 75%^3^ | LC | 2.06 | 1.23;3.44 | 2.21 | 1.23;3.95 |
|  | EMMA | 1 (ref) | - | 1 (ref) | - |

Note: ^1^ >75% of provided patient education and physical exercise LC 63.1% (n=168) EMMA 38.3% (n=95) ^2^ >75% of provided patient education sessions LC 59.7% (n=159) EMMA 37.5% (n=93) ^3^ >75% of provided physical exercise sessions LC 68.7% (n=183) EMMA 51.6% (n=128). ^4^ Adjusted for sex, age, socioeconomic status (living alone, level of education and employed), comorbidities - using Charlson Comorbidity Index, smoking status and level of depression and anxiety (HADS - baseline).
