# Supplementary material for: The patient education strategy “learning and coping” improves adherence to cardiac rehabilitation in primary healthcare settings: a pragmatic cluster-controlled trial
Source: BMC Cardiovasc Disord. 2022 Aug 8;22:364. doi: 10.1186/s12872-022-02774-8 (PMC9361528; doi:10.1186/s12872-022-02774-8)
Supplement: Supplementary file 2 — Additional file 2. Sensitivity analysis including missing HADS responses (LC n=42, EMMA n=19): 20% scored low symptoms (score 0-7), 60% scored moderate symptoms (score 8-10) and 20% scored high symptoms (score 11-21). [file 12872_2022_2774_MOESM2_ESM.docx]

Additional file 2

Table with results from the sensitivity analysis including worst-case scenario, which was tested in a multiple regression model.

|  |  | Crude (N=514) | | Adjusted^1^ (N=514 ) | |
| --- | --- | --- | --- | --- | --- |
|  |  | OR | 95% CI | OR | 95% CI |
| Completing | LC | 1.04 | 0.57;1.89 | 1.13 | 0.71;1.85 |
|  | EMMA | 1 (ref) | - | 1 (ref) | - |

Note ^1^ adjusted for sex, age, socioeconomic status (living alone, level of education and employed), comorbidities - using Charlson Comorbidity Index, smoking status and level of depression and anxiety (HADS - baseline).
